# Fermentation Time Modulates the Osteoprotective Effects of Milk Kefir in Glucocorticoid‐Induced Osteoporotic Rats

**DOI:** 10.1002/mnfr.70601

**Published:** 2026-09-07

**Authors:** Thays Allane Cordeiro Maia, Marco Gabriel Silva Leitão, Everton Cavalcante da Silva, Lorena Vasconcelos Vieira, Sofia Tavares Bessa, Igor Santos da Rocha, Paulo Goberlânio de Barros Silva, Francisca Andrea da Silva Oliveira, Paula Goes, Paola Gyuliane Gonçalves, Deborah Catharine de Assis Leite, Fabio Wildson Gurgel Costa, Lidiany Karla Azevedo Rodrigues, Delane Viana Gondim

**Affiliations:** ^1^ Postgraduate Program in Translational Medicine Faculty of Medicine Federal University of Ceará Fortaleza Ceará Brazil; ^2^ Postgraduate Program in Morphofunctional Sciences Faculty of Medicine Federal University of Ceará Fortaleza Ceará Brazil; ^3^ Postgraduate Program in Dentistry Faculty of Pharmacy Dentistry and Nursing Federal University of Ceará Fortaleza Ceará Brazil; ^4^ Dentistry Course Faculty of Pharmacy Dentistry and Nursing Federal University of Ceará Fortaleza Ceará Brazil; ^5^ Dentistry Course University Center Christus Fortaleza Ceará Brazil; ^6^ Department of Biology Federal University of Ceará Fortaleza Ceará Brazil; ^7^ Specialization Program in Molecular Biology Applied to Bioinformatics Federal Technological University of Paraná Dois Vizinhos Paraná Brazil

**Keywords:** bone remodeling, glucocorticoid, kefir, mandible, osteoporosis

## Abstract

Milk kefir has shown osteoprotective potential, but whether fermentation duration influences its effects remains unclear. This study evaluated the influence of fermentation time on milk kefir microbial composition and its effects on mandibular bone in glucocorticoid‐induced osteoporotic rats. Milk kefir was fermented for 1, 4, or 7 days (MK1, MK4, MK7). Forty male Wistar rats were assigned to five groups (*n* = 8): healthy control, glucocorticoid‐induced osteoporosis (GIO), and GIO treated with MK1, MK4, or MK7. Osteoporosis was induced by weekly intramuscular dexamethasone (7 mg/kg) for 5 weeks, followed by 12 weeks of daily kefir administration (0.7 mL). Microbial composition was assessed by 16S and 18S rRNA gene amplicon sequencing. *Lactobacillus* dominated the bacterial community, whereas *Saccharomyces* predominated among fungi, with no major changes across fermentation times. Prolonged fermentation (MK4 and MK7) significantly increased serum calcium, mandibular cortical thickness, interradicular bone area, collagen maturity, and fracture resistance. These findings indicate that prolonged fermentation enhances the osteoprotective effects of milk kefir, despite relative stability of the dominant microbial taxa, highlighting fermentation duration as an important factor in optimizing milk kefir for preserving mandibular bone under osteoporotic conditions.

## Graphical abstract

Milk kefir exerts osteoprotective effects in glucocorticoid‐induced osteoporotic rats, particularly when fermented for longer periods, improving mandibular bone quality. Fermentation time modulates its microbiological and bioactive profile, highlighting its potential as a functional food for restoring bone health.

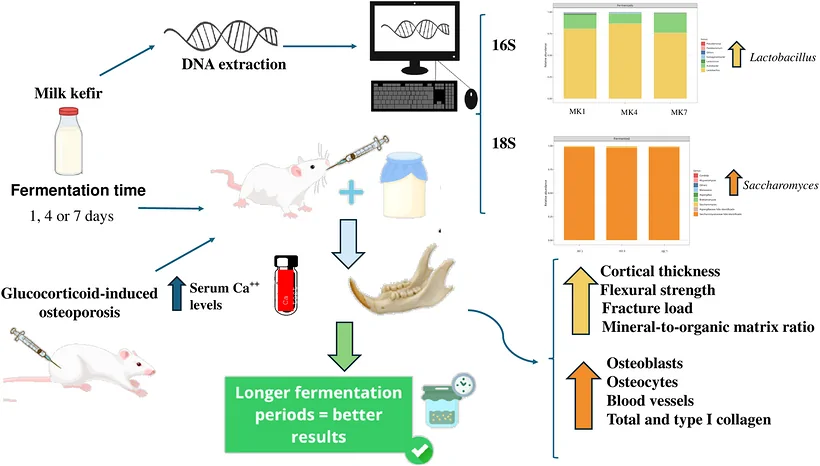

**Abbreviations**
ASVs: amplicon sequence variants
BMD: bone mineral density
C: control group
DNA: deoxyribonucleic acid
EDTA: ethylenediaminetetraacetic acid
GCs: glucocorticoids
GIO: glucocorticoid‐induced osteoporosis
GIO+MK1: animals with glucocorticoid‐induced osteoporosis treated with milk kefir fermented for 1 day
GIO+MK4: animals with glucocorticoid‐induced osteoporosis treated with milk kefir fermented for 4 days
GIO+MK7: animals with glucocorticoid‐induced osteoporosis treated with milk kefir fermented for 7 days
MK: milk kefir
mRNA: messenger ribonucleic acid
PCR: polymerase chain reaction
ROIs: regions of interest
rRNA: ribosomal ribonucleic acid
SEM: standard error of the mean

## 1 Introduction

Osteoporosis is the most prevalent metabolic bone disease, affecting approximately 200 million people worldwide. It is characterized by reduced bone mineral density (BMD) and progressive deterioration of bone microarchitecture due to an imbalance in bone remodeling. These alterations are associated with an increased risk of fractures, which can lead to significant health complications and reduced quality of life [1, 2].

The condition most frequently affects the lumbar spine, with prevalence rates ranging from 15% to 38%, and to a lesser extent the femoral neck, with rates between 10% and 18% [3]. However, the rising prevalence of osteoporosis highlights the need for attention to its potential oral and maxillofacial manifestations [4]. Recent studies have shown that osteoporosis can also affect bones with a high trabecular content, such as the mandible [3, 4].

Osteoporotic changes in the mandible include alveolar bone height loss, increased marrow spaces, thinning of trabecular bone, erosion of the mandibular cortex, and reduced BMD [3]. These morphological alterations are associated with various oral complications, including increased tooth loss, worsening of periodontal disease, impaired osseointegration of dental implants, failure of orthodontic treatments, higher fracture risk, and delayed bone healing after surgical procedures due to compromised bone quantity and quality [3, 4].

Glucocorticoid‐induced osteoporosis (GIO) is the most common form of secondary osteoporosis. Glucocorticoids (GCs) are widely used in the treatment of various diseases [5] due to their anti‐inflammatory and immunosuppressive properties, with hydrocortisone, prednisone, and dexamethasone being the most commonly used agents in clinical practice [6, 7]. However, prolonged use of GCs at high doses leads to multiple adverse metabolic effects, including impaired bone remodeling [8].

Current therapeutic strategies for osteoporosis aim to reduce bone resorption and improve BMD and strength. Calcium supplementation is widely recommended, given its essential role in bone formation and maintenance [9]. The use of probiotics as calcium delivery vehicles has attracted scientific interest for the management of various diseases, including osteoporosis [10].

Milk kefir (MK) is a fermented product resulting from the symbiotic association of bacteria and yeasts present in kefir grains [11]. It has high nutritional value, being a rich source of proteins and calcium [12]. Although traditionally prepared with 24 h of fermentation, studies suggest that longer fermentation times increase microbial diversity and may enhance its health benefits [13, 14, 15].

However, despite the reported benefits of MK on bone tissue, no studies to date have evaluated its effects on the mandible in the context of GIO. Therefore, the present study aimed to assess the protective effect of MK would be modulated by fermentation time and microbial variety.

## 2 Experimental Section

### 2.1 DNA Extraction and Data Analysis of 16S and 18S Ribosomal RNA (rRNA) Gene Amplicons

MK grains were obtained from the Dairy Laboratory of the Federal University of Ceará. The kefir beverage was prepared by inoculating 5% (*w*/*v*) kefir grains into sterilized milk. After incubation at 25°C–28°C for 1, 4, or 7 days, the grains were separated from the fermented milk by filtration using a plastic strainer [14].

MK samples were collected after 1, 4, and 7 days of fermentation, and total DNA was extracted using the DNeasy PowerSoil Pro Kit (Qiagen, Hilden, Germany) according to the manufacturer's instructions.

For microbial profiling, the V4 region of the bacterial 16S rRNA gene was amplified using primers 515F‐Y and 806R [16, 17], while the V9 region of the eukaryotic 18S rRNA gene was amplified using primers EUK1391‐F and euk‐Br [18]. Each polymerase chain reaction (PCR) reaction (30 µL final volume) contained 2 µL of genomic DNA (5 ng/µL), 0.75 µL of each primer (10 µM), 6.0 µL of GoTaq G2 Hot Start buffer (5×, Promega), 3.6 µL of MgCl_2_ (25 mM, Promega), 0.6 µL of dNTPs (10 mM, Promega), 0.2 µL of Taq polymerase (5 U/µL, Promega), and 16.1 µL of nuclease‐free ultrapure water (Promega, Madison, WI, USA). PCR amplification was carried out in an Eppendorf Mastercycler Gradient (Eppendorf, Hamburg, Germany) under the following program: initial denaturation at 95°C for 3 min; 35 cycles of denaturation at 98°C for 30 s, annealing at 55°C for 30 s, and extension at 72°C for 45 s; followed by a final extension at 72°C for 5 min. PCR products were verified on 2% (*w*/*v*) agarose gels and purified using AMPure XP beads (Beckman Coulter, Brea, CA, USA).

Indexed libraries were generated in a second PCR by adding Illumina adapters, using a reaction mixture containing 25 µL of 2× KAPA HiFi ReadyMix (Roche), 5 µL of each Nextera XT index (Illumina, San Diego, CA, USA), 5 µL of purified amplicon, and 10 µL of nuclease‐free water. PCR products were purified again with AMPure XP beads. DNA concentration was determined using a Qubit 2.0 fluorometer (Invitrogen, Carlsbad, CA, USA), and libraries were normalized following Illumina's guidelines. Equimolar amounts were pooled, diluted, and denatured before sequencing on an Illumina MiSeq platform using the V2 Reagent Kit (300 cycles, Illumina, USA).

Bioinformatic analyses were performed using QIIME 2 (version 2023.2). Raw reads were processed through the q2‐DADA2 pipeline for quality filtering, dereplication, and chimera removal, yielding amplicon sequence variants (ASVs). Taxonomic assignment was carried out by aligning representative ASVs against the SILVA reference database (full‐length 16S rRNA). Reads corresponding to singletons, chloroplasts, mitochondria, or archaea were removed during curation. Relative abundance of microbial taxa was analyzed using the MicrobiomeAnalyst 2.0 platform (https://www.microbiomeanalyst.ca/).

### 2.2 Animals

All animal procedures were approved by the Animal Ethics Committee of the Federal University of Ceará, under protocol number 34300822‐0. All experiments were conducted in accordance with the ARRIVE guidelines and the Guide for the Care and Use of Laboratory Animals. The animals were housed in polypropylene cages in a temperature‐controlled environment (22°C), with a 12‐h light/dark cycle, and had free access to food and water.

Sample size calculation was performed considering the animal as the experimental unit, with histomorphometric analysis as the primary endpoint. The sample size was determined to provide 80% power to detect a 20% difference between groups, assuming a standard deviation of 15% and a 95% confidence interval (*p* = 0.05). As a result, eight animals were included in each experimental group.

Forty male Wistar rats (*Rattus norvegicus albinus*), aged 60 days and weighing approximately 200 g, were animals randomly divided into the following experimental groups (*n* = 8 per group): Control (C; healthy animals without osteoporosis that received only saline solution (0.7 mL/animal/day); Osteoporosis: animals with GIO that received saline solution (0.7 mL/animal/day) or MK fermented for 1, 4, or 7 days (GIO+MK1, GIO+MK4, GIO+MK7, respectively). The osteoporotic animals received saline solution or MK daily by oral gavage for 12 weeks.

### 2.3 GIO Induction

**FIGURE 1:**
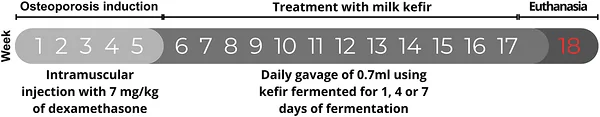
Experimental protocol.

Osteoporosis was induced by intramuscular injection of dexamethasone (Decadron; 4 mg/mL) at a dose of 7 mg/kg, once a week for 5 consecutive weeks [19] (Figure 1).

### 2.4 MK Fermentation

Fermentation timepoints of 1, 4, and 7 days were selected to capture distinct stages of MK maturation. The 1‐day timepoint represents the early phase of microbial succession and acidification. Although most artisanal fermentations are typically completed within 1–2 days at room temperature, extended fermentation periods can occur under lower temperatures or when a more acidic flavor is desired. Therefore, 4 days was chosen as an intermediate point, reflecting a partially matured product in which microbial composition and metabolite accumulation begin to change substantially. The 7‐day timepoint represents a fully matured fermentation, in which selective enrichment of taxa such as *Lactobacillus kefiri* and the accumulation of bioactive metabolites are more pronounced. These intervals also align with our preliminary Raman spectroscopy data, which indicated a progressive increase in *L. kefiri* with longer fermentation, supporting the investigation of time‐dependent effects on microbial dynamics and functional bioactivity [14].

### 2.5 Dosage Regimen

After preparation of MK fermented for 1, 4, or 7 days, the beverage (0.7 mL/day) was administered by oral gavage at the same time each morning [14]. The treatment lasted 12 weeks and began one week after completion of the GIO protocol [9] (Figure 1). This regimen corresponds to an estimated intake of approximately 3.5 g/kg body weight/day of kefir solids, equivalent to a human equivalent dose of ∼0.6 g/kg/day, assuming a standard allometric scaling factor (Km = 6 for rats; 37 for humans). This dose is achievable through regular dietary consumption of fermented milk beverages. The selected dosage and duration were based on previous studies reporting systemic and bone‐protective effects of MK without adverse outcomes [9, 14].

### 2.6 Euthanasia and Sample Collection for Analysis

At week 18 of the experiment, all animals were anesthetized with a combination of xylazine hydrochloride 2% (10 mg/kg) and ketamine hydrochloride 5% (80 mg/kg) (Figure 1). Blood samples were collected for serum calcium analysis. From each animal, 1 mL of blood was obtained by cardiac puncture, followed by euthanasia through exsanguination. The collected blood was placed in microtubes containing 20 µL of sodium heparin as an anticoagulant, gently homogenized, kept refrigerated, and then centrifuged at 3000 rpm for 15 min. The resulting plasma was stored at –80°C until serum calcium determination.

Mandibles were dissected, and the right hemimandibles were fixed in 10% buffered formalin for 24 h for subsequent histological processing [20]. The left hemimandibles were stored at –80°C for later radiographic, biomechanical, and Raman spectroscopy analyses.

### 2.7 Serum Calcium Analysis

Plasma samples were thawed at room temperature prior to analysis. Serum calcium levels were measured according to the manufacturer's instructions (Bioclin, Belo Horizonte, Brazil) using an automated spectrophotometer (BM 200, Vyttra, São Paulo, Brazil). The absorbance of the reaction product was measured at wavelengths between 600 and 680 nm.

### 2.8 Radiographic Analysis

The left hemimandibles were radiographed alongside a grayscale reference scale using a dental x‐ray (Dabi Atlante, Spectro 70X, Class I, Type B) operating at 70 kVp and 8 mA, with a standardized focus‐to‐object distance of 30 cm and an exposure time of 250 ms. The hemimandibles were positioned with the lingual surface in contact with the active side of the digital sensor, and the x‐ray beam was directed perpendicularly to the sensor surface [21, 22].

Radiodensity was assessed in two specific regions of interest (ROIs): (1) the inferior cortical bone and (2) the interradicular bone of the left mandibular first molar, using ImageJ software (La Crosse, WI, USA). The interradicular region was defined as the area between the periodontal ligament spaces of the roots of the first molar and the lower incisor, extending from the apex of the distal root of the first molar to the mesial alveolar bone of this tooth [22] (Figure SM1).

To normalize radiodensity values, an 11‐step grayscale reference (densitometer) was used for calibration. Radiodensities were measured at steps 1, 6, and 11, as well as at a radiolucent point on the film. These values were used to generate a linear equation for each radiograph, which was then applied to normalize the radiodensity measurements of the selected ROIs.

Cortical bone thickness was measured at three standardized points along the inferior border of the mandible: directly below the emergence point of the incisor; at the most posterior point, corresponding to the mandibular angle; and at a midpoint between these two anatomical landmarks [22] (Figure SM1). Measurements were performed using Inkscape software (Boston, MA, USA). Additionally, the interradicular bone area was quantified using ImageJ software (La Crosse, WI, USA).

### 2.9 Three‐Point Bending Test

Vestibulolingual thickness and height at the center of the mandibular first molar were measured using a digital caliper. These measurements were entered into the Bluehill 2 software (Instron Corp., USA) prior to each test.

The left hemimandibles were positioned with the buccal surface facing upward, supported on two fixtures placed 10 mm apart, with the central loading point aligned at the midpoint of the left mandibular first molar. During the bending test, a 500 N load cell was applied at a constant speed of 5 mm/min until fracture occurred [23].

From the load–displacement curves, the following biomechanical parameters were calculated: fracture load and flexural strength [23, 24, 25].

### 2.10 Raman Spectroscopy Analysis

Sample preparation was performed using a metallographic cutter (Isomet 4000, Buehler, Uzwil, Switzerland) equipped with a diamond blade, under distilled water irrigation and low speed (100 rpm), to section the samples and expose the area of interest. The left hemimandibles were sectioned in the buccolingual direction at the center of the first molar region. Additional parallel cuts were made to facilitate sample positioning with the surface perpendicular to the laser beam.

The exposed surfaces were polished at the region of interest using a sequential series of abrasive papers (500, 600, and 1200 grit) under irrigation, for 30 s each. The samples were then placed in an ultrasonic bath for 10 min to remove debris [26].

Raman spectra were acquired using an argon ion laser with a 532 nm wavelength, operating at 25% power and 100× magnification. Spectra were collected over a range of 400–1800 cm^−^^1^ with a total acquisition time of 60 s, consisting of three accumulations of 20 s each [27]. Based on the intensity of the Raman peaks, the following parameters were analyzed: mineral‐to‐matrix ratio, collagen maturity (pyridinoline/dehydro‐dihydroxylysinonorleucine ratio, proteoglycan content, type B carbonate substitution, and mineral maturity or crystallinity [28, 29].

### 2.11 Histomorphometric Analysis

The samples of the right hemimandibles were fixed in 10% buffered formalin for 24 h and then decalcified in 10% ethylenediaminetetraacetic acid (EDTA) at pH 7.4 for 60 days at room temperature. Following decalcification, the samples were dehydrated in absolute ethanol, cleared in xylene, and embedded in paraffin. Sections (10 µm thick) were obtained in the mesiodistal direction across the molar region [30]. The slides were stained with hematoxylin and eosin (H and E) and analyzed under light microscopy (Leica DM 2000, Wetzlar, Germany).

The histomorphometric analysis was restricted to the interradicular bone region of the mandible first molar (Figure SM2), consistently defined across all samples and predominantly composed of trabecular bone. Within this region, five randomly non‐overlapping fields were selected per sample using a standardized approach and analyzed by an examiner blinded to group allocation.

The slides were analyzed at 400× magnification to count the number of empty osteocyte lacunae, viable osteocytes, blood vessels per bone area, and osteoblasts per bone perimeter [31]. All standardized measurements were performed using an image analysis system (ImageJ software, La Crosse, WI, USA).

### 2.12 Total and Type I Collagen Quantification

To determine the amount and type of collagen, the 6 µm‐thick sections were obtained from the interradicular bone region of the mandibular first molar using previously prepared paraffin blocks. The sections were stained with picrosirius red and evaluated under both bright‐field and polarized light microscopy for quantification of total collagen and Type I collagen (yellow‐red birefringence), respectively. For each sample, five non‐overlapping fields were selected at 400× magnification using a binocular microscope (Leica DM 2000, Wetzlar, Germany) following a systematic random sampling approach within the interradicular region. Quantitative analysis was performed on photomicrographs taken before and after light polarization, using ImageJ software (La Crosse, WI, USA) [32].

### 2.13 Statistical Analysis

Normality and homogeneity of variance were assessed using the Shapiro–Wilk and Levene tests, respectively. Parametric data were expressed as means ± standard error of the mean (SEM). One‐way analysis of variance (ANOVA) followed by Tukey's post hoc test was used for group comparisons. A *p* value < 0.05 was considered statistically significant. All statistical analyses were performed using GraphPad Prism 8 software.

## 3 Results

### 3.1 Microbial Taxonomic Composition of MK Across Fermentation Time

**FIGURE 2:**
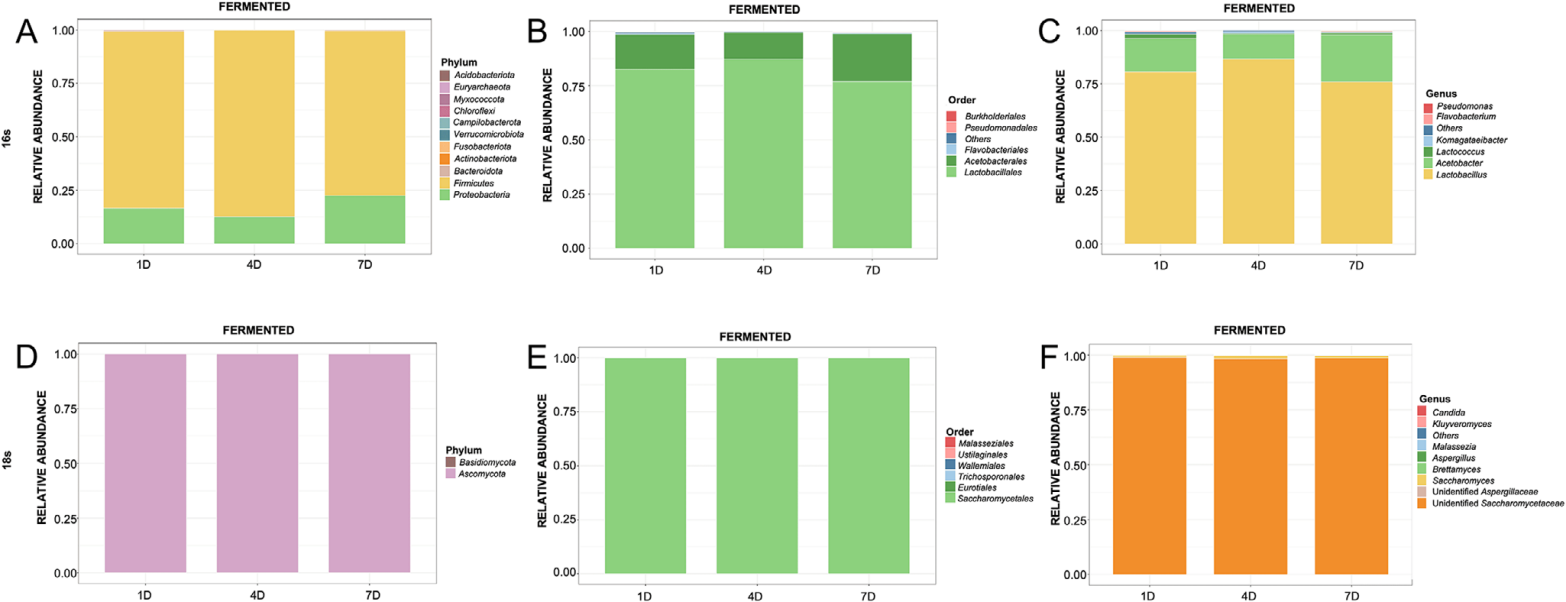
Relative bacterial and yeast abundance in fermented milk kefir at 1, 4, and 7 days, presented at both the phylum (A and D), order (B and E), and genus (C and F) levels. 1D, milk kefir fermented for 1 day; 4D, milk kefir fermented for 4 days; 7D, milk kefir fermented for 7 days.

The taxonomic analysis of MK revealed that the bacterial community was strongly dominated by the phylum Firmicutes, which remained relatively stable over time, showing only minor fluctuations in abundance during fermentation. Firmicutes represented approximately 85% of the bacterial taxa after 1 day, increased slightly to 87% at 4 days, and decreased to around 80% after 7 days of fermentation (Figure 2A).

At the genus level, *Lactobacillus* (order Lactobacillales) was consistently the most abundant taxon, comprising approximately 80% of bacterial sequences at 1 day, peaking at 85% after 4 days, and remaining predominant at about 76% after 7 days (Figure 2B,C). The order Acetobacterales was the second most prevalent group, contributing roughly 17%, 12%, and 22% at 1, 4, and 7 days, respectively (Figure 2B).

In contrast, the fungal community displayed an even higher level of stability, with the phylum Ascomycota accounting for approximately 100% of the detected fungal taxa at all fermentation times (Figure 2D). Members of the family *Saccharomycetaceae* (order Saccharomycetales) were consistently predominant across all time points, reflecting a highly stable fungal community dominated by yeast‐like organisms typically associated with kefir fermentation (Figure 2E,F).

### 3.2 Longer MK Fermentation Increases Serum Calcium in Rats With GIO

**FIGURE 3:**
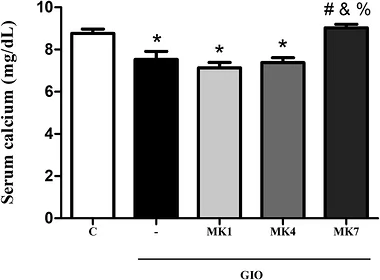
Serum calcium levels in osteoporotic rats treated with milk kefir fermented for 1, 4, or 7 days. Results are expressed as mean ± SEM from biological replicates (*n* = 8 animals per group). *p < 0.05 vs. C; # *p* < 0.05 vs. GIO; and *p* < 0.05 vs. GIO+MK1; % *p* < 0.05 vs. GIO+MK4. One‐way ANOVA followed by Tukey's post hoc test. C, control; GIO, glucocorticoid‐induced osteoporosis; MK1, milk kefir treatment fermented for 1 day; MK4, milk kefir treatment fermented for 4 days; MK7, milk kefir treatment fermented for 7 days.

A significant reduction in serum calcium levels was observed in the GIO (*p* = 0.016), GIO+MK1 (*p* = 0.001), and GIO+MK4 (*p* = 0.006) groups compared to the C group. In contrast, rats treated with milk kefir fermented for 7 days (GIO+MK7) exhibited a significant increase in serum calcium levels compared to the GIO (*p* = 0.002), GIO+MK1 (*p* = 0.0002), and GIO+MK4 (*p* = 0.001) groups. Notably, calcium levels in the GIO+MK7 group did not differ significantly from those in the C group (Figure 3).

### 3.3 MK Fermentation Improves Mandibular Bone Structural Parameters in Osteoporotic Rats

**FIGURE 4:**
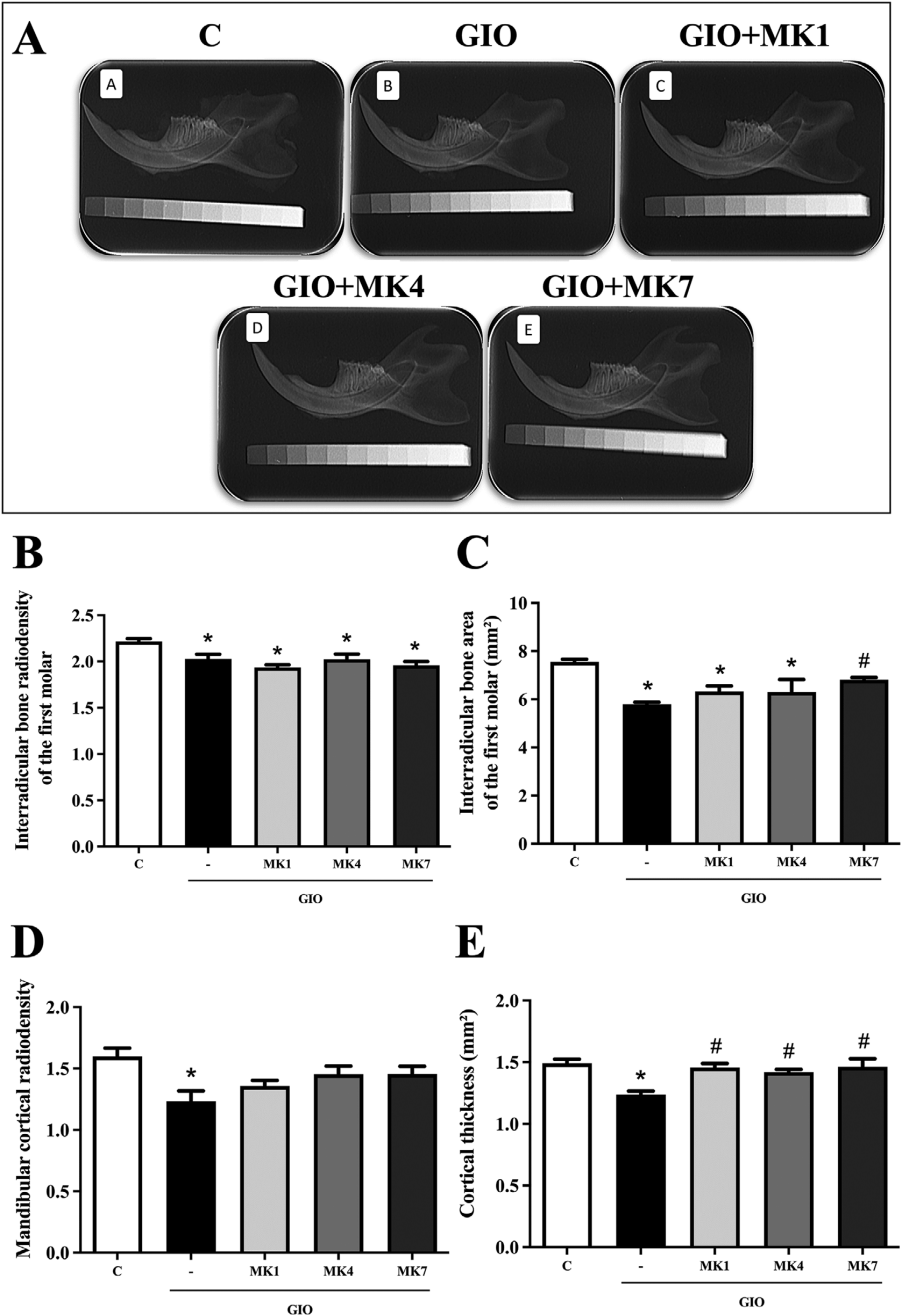
(A) Radiographic images of the mandible in osteoporotic rats treated with milk kefir fermented for 1, 4, or 7 days. Graphs show the quantification of interradicular bone radiodensity (B) and bone area (C) of the lower first molar, as well as mandibular cortical bone radiodensity (D) and cortical bone thickness (E). Results are expressed as mean ± SEM from biological replicates (*n* = 8 animals per group). *p < 0.05 vs. C; # *p* < 0.05 vs. GIO. One‐way ANOVA followed by Tukey's post hoc test. C, control; GIO, glucocorticoid‐induced osteoporosis; MK1, milk kefir treatment fermented for 1 day; MK4, milk kefir treatment fermented for 4 days; MK7, milk kefir treatment fermented for 7 days.

Representative radiographs of the mandible are shown in Figure 4A. In the interradicular region of the left mandibular first molar, bone radiodensity was significantly reduced in the GIO (*p* = 0.017), GIO+MK1 (*p* = 0.0001), GIO+MK4 (*p* = 0.014), and GIO+MK7 (*p* = 0.0004) groups compared with the C group, with no significant differences among the osteoporotic groups (Figure 4B). Interradicular bone area was reduced in GIO (*p* = 0.0001), GIO+MK1 (*p* = 0.004), and GIO+MK4 (*p* = 0.004) compared with C, whereas only GIO+MK7 showed a significant increase compared with GIO (*p* = 0.014), indicating that prolonged fermentation was necessary to improve this parameter (Figure 4C).

Mandibular cortical bone radiodensity was decreased in GIO compared with C (*p* = 0.008), with no differences among kefir‐treated groups (Figure 4D). Cortical thickness was reduced in GIO (*p* = 0.001) but significantly increased in all kefir‐treated groups (GIO+MK1, *p* = 0.004; GIO+MK4, *p* = 0.027; GIO+MK7, *p* = 0.005) compared with GIO, indicating that even shorter fermentation durations improved this parameter (Figure 4E).

### 3.4 MK Fermented for 4 and 7 Days Improves Fracture Load and Flexural Strength in Osteoporotic Rats

**FIGURE 5:**
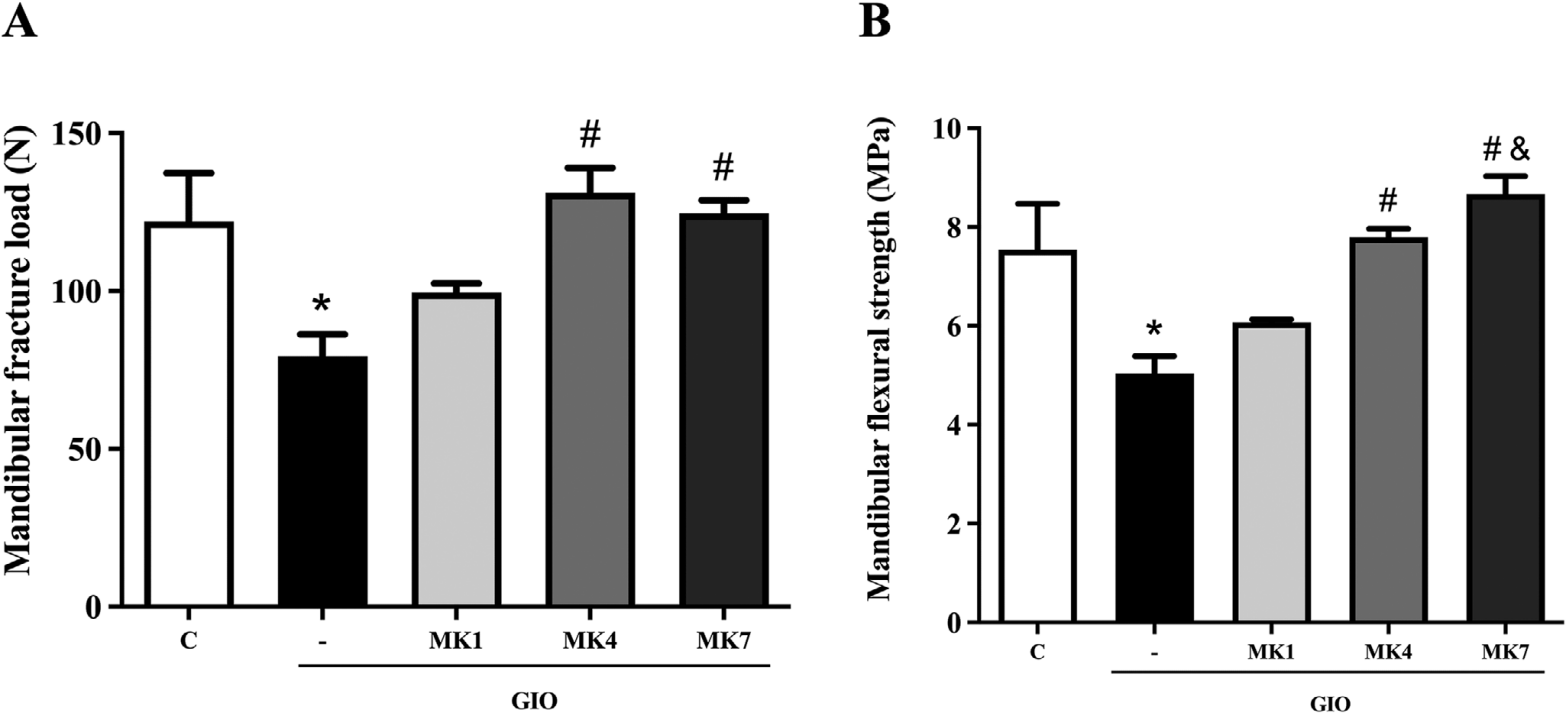
Quantification of mandibular fracture load (A) and flexural strength (B) in osteoporotic animals treated with milk kefir fermented for 1, 4, or 7 days. Results are expressed as mean ± SEM from biological replicates (*n* = 8 animals per group). *p < 0.05 vs. C; # *p* < 0.05 vs. GIO; and *p* < 0.05 vs. GIO+MK1. One‐way ANOVA followed by Tukey's post hoc test. C, control; GIO, glucocorticoid‐induced osteoporosis; MK1, milk kefir treatment fermented for 1 day; MK4, milk kefir treatment fermented for 4 days; MK7, milk kefir treatment fermented for 7 days.

Fracture load and mandibular flexural strength were significantly reduced in animals from the GIO group (*p* = 0.014 and *p* = 0.005, respectively) compared to the C group (Figure 5A,B). In contrast, treatment with milk kefir fermented for 4 days (GIO+MK4; *p* = 0.002) and 7 days (GIO+MK7; *p* = 0.008 and *p* = 0.0001, respectively) significantly improved these mechanical properties relative to the GIO group (Figure 5A,B). Furthermore, flexural strength was significantly higher in the GIO+MK7 group compared to the GIO+MK1 group (*p* = 0.002) (Figure 5B).

### 3.5 MK Fermented for 4 and 7 Days Improves Bone Composition and Collagen Levels in Mandibles of Osteoporotic Rats

Mandibular bone analysis revealed that osteoporotic animals had significantly reduced mineral‐to‐organic matrix ratio (*p* = 0.007), collagen maturity (*p* = 0.017), and proteoglycan content (*p* = 0.001) compared to the C group. Treatment with MK fermented for 4 or 7 days restored these parameters (4 days: *p* = 0.002, *p* = 0.012, *p* = 0.001; 7 days: *p* = 0.009, *p* = 0.044, *p* = 0.010) relative to GIO animals (Table 1). No differences were observed for type B carbonate substitution or crystallinity (Table 1).

**TABLE 1:** Mandibular bone composition measurements by Raman spectroscopy in rats with glucocorticoid‐induced osteoporosis treated with milk kefir fermented for 1, 4, or 7 days.

Parameters	C	GIO	GIO+MK1	GIO+MK4	GIO+MK7
Groups					
Mineral/organic matrix ratio	2.885 ± 0.296	1.884 ± 0.114^a^	2.221 ± 0.134	2.986 ± 0.227^b^	2.858 ± 0.101^b^
Collagen maturity	1.098 ± 0.005	1.047 ± 0.006^a^	1.067 ± 0.011	1.097 ± 0.010^b^	1.094 ± 0.017^b^
Relative proteoglycan content	0.942 ± 0,010	0.877 ± 0.002^a^	0.921 ± 0.012	0.949 ± 0.017^b^	0.936 ± 0.008^b^
Type B carbonate substitution	0.520 ± 0.042	0.552 ± 0.046	0.524 ± 0.026	0.556 ± 0.078	0.497 ± 0.030
Crystallinity	1.040 ± 0.013	1.041 ± 0.007	1.023 ± 0.007	1.026 ± 0.010	1.017 ± 0.009

*Note*: One‐way ANOVA followed by Tukey's post hoc test.

**Abbreviations**: C, control; GIO, glucocorticoid‐induced osteoporosis; MK1, milk kefir treatment fermented for 1 day; MK4, milk kefir treatment fermented for 4 days; MK7, milk kefir treatment fermented for 7 days.

^a^ *p* < 0.05 versus C.

^b^ *p* < 0.05 versus GIO.

**FIGURE 6:**
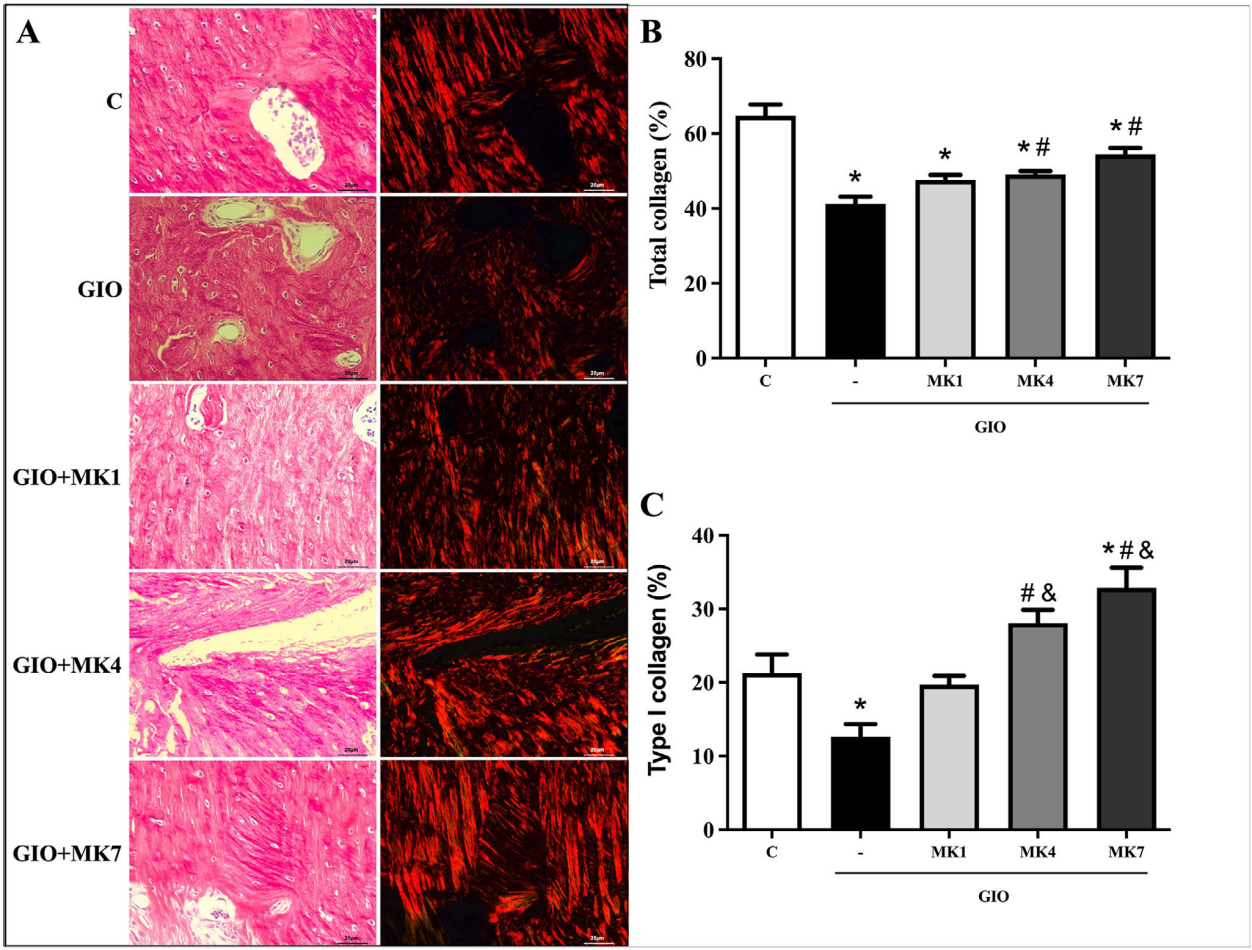
(A) Photomicrographs of collagen fibers in the interradicular bone region of the left mandibular first molar in osteoporotic rats treated with milk kefir, stained with Picrosirius Red. Left column: bright‐field microscopy (non‐polarized light); right column: polarized light microscopy. Images were obtained at 400× magnification. (B) Total collagen content. (C) Type I collagen content. Images were obtained at 400× magnification. Results are expressed as mean ± SEM from biological replicates (*n* = 8 animals per group). *p < 0.05 vs. C; # *p* < 0.05 vs. GIO; and *p* < 0.05 vs. GIO+MK1. One‐way ANOVA followed by Tukey's post hoc test. C, control; GIO, glucocorticoid‐induced osteoporosis; MK1, milk kefir treatment fermented for 1 day; MK4, milk kefir treatment fermented for 4 days; MK7, milk kefir treatment fermented for 7 days.

Total collagen was significantly reduced in all GIO groups versus C (*p* = 0.0001), but was increased in GIO+MK4 (*p* = 0.016) and GIO+MK7 (*p* = 0.0002) compared with GIO (Figure 6A,B). Type I collagen was also reduced in GIO (*p* = 0.029); treatment with MK fermented for 4 or 7 days significantly increased type I collagen compared with both GIO (*p* = 0.0001) and GIO+MK1 (*p* = 0.038 and *p* = 0.001, respectively). Notably, GIO+MK7 showed higher type I collagen than C (*p* = 0.006) (Figure 6A,C).

### 3.6 MK Improves Mandibular Bone Histomorphometric Parameters and Vascularization in Osteoporotic Rats

**FIGURE 7:**
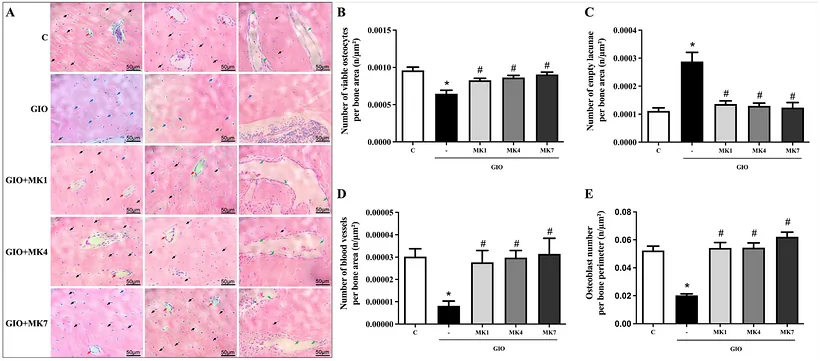
Photomicrographs (A) and quantification of the number of viable osteocytes (B), empty lacunae (C), blood vessels(D) per area, and osteoblasts per perimeter (E) in the interradicular bone region of the left mandibular first molar in osteoporotic rats treated with milk kefir. Images were obtained at 400× magnification. Results are expressed as mean ± SEM from biological replicates (*n* = 8 animals per group). *p < 0.05 vs. C; # *p* < 0.05 vs. GIO. One‐way ANOVA followed by Tukey's post hoc test. C, control; GIO, glucocorticoid‐induced osteoporosis; MK1, milk kefir treatment fermented for 1 day; MK4, milk kefir treatment fermented for 4 days; MK7, milk kefir treatment fermented for 7 days.

Histomorphometric evaluation of the mandible (Figure 7A) revealed a significant reduction in the number of viable osteocytes (*p* = 0.0001) and blood vessels (*p* = 0.010) per bone area in the GIO group compared to the control group (Figure 7B,D). A significant decrease in the number of osteoblasts per bone perimeter was also observed in the GIO group (*p* = 0.0001) compared to the C group (Figure 7E).

However, animals treated with MK, regardless of fermentation time (GIO+MK1, GIO+MK4, and GIO+MK7), showed a significant increase in the number of viable osteocytes (*p* = 0.010, *p* = 0.002, and *p* = 0.0004, respectively), blood vessels (*p* = 0.027, *p* = 0.018, and *p* = 0.010), and osteoblasts (*p* = 0.0001) compared to the GIO group. No significant differences were found among the MK‐treated groups (Figure 7B,D,E).

GIO also significantly increased the number of empty lacunae per bone area compared to the C group (*p* = 0.0001). MK treatment at all fermentation times significantly reduced the number of empty lacunae compared to the GIO group (*p* = 0.0001) (Figure 7C).

Importantly, none of the MK‐treated groups differed significantly from the C group in any of the evaluated parameters, suggesting a restoration of normal mandibular bone histomorphometry.

## 4 Discussion

This study demonstrated that both bacterial and fungal communities in MK were dominated by a single major taxon. While the bacterial composition showed slight temporal variations, the fungal community remained remarkably stable. Kefir fermented for extended periods was associated with improved mandibular bone quality in GC‐treated rats. Although a causal relationship cannot be firmly established, these findings suggest that fermentation duration modulates kefir's functional properties and enhances its probiotic potential.

Prolonged fermentation influenced both microbial composition and biological activity. In our previous study, extending fermentation from 1 to 7 days markedly altered kefir's microbial profile and biological effects, promoting enrichment of *Lactobacillus* species, particularly *L. kefiri* [14]. This shift may have contributed to improved kefir's bioactivity, as *Lactobacillus* spp. produce metabolites with immunomodulatory and anti‐inflammatory effects that extend beyond the gut, including impacts on bone metabolism [33]. The stable fungal community dominated by *Saccharomycetaceae* may have acted synergistically, since yeast‐derived metabolites have been reported to suppress inflammatory osteoclastogenesis in osteoporosis models.

The mandible, often overlooked in osteoporosis research, undergoes structural and metabolic changes comparable to axial bones under osteoporotic conditions. In this study, GC administration led to significant mandibular bone deterioration, including cortical thinning, reduced vascularization, reduced osteocytes within lacunae, and impaired mechanical strength. These alterations highlight the mandible as a sensitive and clinically relevant site for evaluating bone fragility and therapeutic efficacy in experimental osteoporosis.

GCs disrupt calcium homeostasis by impairing intestinal absorption and increasing renal excretion, leading to hypocalcemia and bone loss [34]. Consistent with this, our GIO model exhibited markedly reduced serum calcium levels. Treatment with kefir fermented for seven days increased serum calcium levels toward control values. This effect may reflect improved calcium absorption and bioavailability, as previously suggested in the literature [33, 35], although the specific mechanisms underlying this response were not directly investigated in the present study. In addition to calcium, kefir provides magnesium, zinc, and bioactive proteins [33], which may synergistically contribute to bone mineralization and attenuation of bone loss [36].

From a biomechanical perspective, GIO significantly reduced fracture resistance, in agreement with previous studies using ovariectomized or GC‐based models [23, 37]. Kefir treatment improved mechanical strength, with more pronounced effects after 4‐ and 7‐day fermentation. This improvement may be associated with the accumulation of bioactive peptides and metabolites generated during extended fermentation, as suggested in previous studies [15, 38]. Further investigations are warranted to characterize kefir's peptide profiles and identify specific strains or compounds responsible for these effects.

Raman spectroscopy and collagen analyses confirmed major alterations in osteoporotic bone, including decreased mineral‐to‐matrix ratio, proteoglycan content, collagen maturity, and reduced levels of total and type I collagen, all consistent with the observed mechanical weakness. These findings are consistent with reports that GCs suppress type I collagen synthesis by downregulating pro‐collagen mRNA and impairing osteoblast function [39, 40]. MK treatment attenuated these alterations, which may be associated with modulation of osteoblast activity and matrix‐regulating pathways [41], although these mechanisms were not directly assessed in the present study.

Our previous work demonstrated that MK fermented for 24 h improved femoral bone composition in GIO, particularly when combined with resistance training [42]. The present study extends these findings to the mandible, showing that mandibular bone is similarly responsive but particularly dependent on longer fermentation durations. This may be related to increased proteolytic activity during fermentation, which has been associated with higher levels of bioactive peptides in previous studies [13, 15].

At the cellular level, MK attenuated osteoporosis‐associated reductions in osteocyte and osteoblast numbers and improved vascularization. GCs markedly reduce bone vascularity, compromising nutrient delivery and promoting apoptosis and autophagy in bone cells [43, 44]. Osteocyte loss further weakens mechanotransduction and bone hydration [42, 45]. The effects of MK on bone cellular parameters and vascularization may be associated with biological pathways related to apoptosis and angiogenesis, as previously described in the literature [46, 47].

This study used only male rats, based on previous evidence that estrogens protect bone metabolism by regulating osteoclast lifespan and preventing apoptosis in bone cells via estrogen receptor α signaling [30, 48]. However, future studies should include female models to assess the potential influence of sex hormones on kefir's osteoprotective effects.

In summary, GIO markedly impaired mandibular bone quality, whereas MK, particularly after 4 or 7 days of fermentation, mitigated these deleterious effects by improving structural, compositional, and mechanical parameters of bone. The metagenomic data revealed a microbial community dominated by *Lactobacillus* and *Saccharomycetaceae*, with subtle time‐dependent shifts that may influence kefir's functionality. Collectively, these findings highlight fermentation duration as a determinant of kefir's biological properties and support its potential as a functional food adjunct for maintaining bone health under osteoporotic conditions.

## Author Contributions

**Thays Allane Cordeiro**: methodology, investigation, formal analysis, writing – original draft, and writing – review and editing. **Marco Gabriel Silva Leitão**: investigation and formal analysis. **Everton Cavalcante da Silva**: investigation. **Lorena Vasconcelos Vieira**: investigation; **Sofia Tavares Bessa**: investigation. **Igor Santos da Rocha**: investigation. **Paulo Goberlânio de Barros Silva**: methodology, formal analysis, and data curation. **Francisca Andrea da Silva Oliveira**: methodology and investigation. **Paula Goes**: methodology and formal analysis. **Paola Gyuliane Gonçalves**: methodology and investigation. **Deborah Catharine de Assis Leite**: methodology and investigation. **Fabio Wildson Gurgel Costa**: methodology and formal analysis. **Lidiany Karla Azevedo Rodrigues**: conceptualization, methodology, and resources. **Delane Viana Gondim**: conceptualization supervision, resources, methodology, and writing – review and editing.

## Funding

The authors have nothing to report.

## Conflicts of Interest

The authors declare no conflicts of interest.

## Supporting information




**Supporting File 1**: mnfr70601‐sup‐0001‐SuppMat.docx.

**Supporting File 2**: mnfr70601‐sup‐0002‐SuppMat.tiff.

**Supporting File 3**: mnfr70601‐sup‐0003‐SuppMat.jpg.

## Data Availability

The data that support the findings of this study are available from the corresponding author upon reasonable request.
