# Supplementary material for: Fermentation Time Modulates the Osteoprotective Effects of Milk Kefir in Glucocorticoid‐Induced Osteoporotic Rats
Source: Mol Nutr Food Res. 2026 Sep 7;70(17):e70601. doi: 10.1002/mnfr.70601 (PMC13548182; doi:10.1002/mnfr.70601)
Supplement: Supplementary file 1 [file MNFR-70-e70601-s001.docx]

Supplementary Material - legends

Figure SM1- Schematic representation of the mandibular regions analyzed. The illustration defines the regions of interest consistently applied across all samples. The areas used for radiographic assessment are highlighted. The interradicular bone region is indicated in black, and the cortical bone region is delineated by a square.

Figure SM2- Schematic representation of the interradicular region analyzed. The interradicular bone region is highlighted in red, indicating the area selected for histological analysis.
